# Gene Mutational Clusters in the Tumors of Colorectal Cancer Patients With a Family History of Cancer

**DOI:** 10.3389/fonc.2022.814397

**Published:** 2022-06-24

**Authors:** He Huang, Ting Deng, Yuntong Guo, Hao Chen, Xiaolong Cui, Jingjing Duan, Yuchong Yang, Zhixin Guo, Yi Ba

**Affiliations:** ^1^ Department of Gastrointestinal Surgery, The First Hospital of Shanxi Medical University, Taiyuan, China; ^2^ Tianjin Medical University Cancer Institute and Hospital, National Clinical Research Center for Cancer, Key Laboratory of Cancer Prevention and Therapy, Tianjin's Clinical Research Center for Cancer, Tianjin, China; ^3^ Gastrointestinal Surgery, Hebei Dingzhou People’s Hospital, Dingzhou, China

**Keywords:** colorectal cancer, mutation, family history, co-mutation, biomarkers

## Abstract

**Introduction:**

Family history is a high-risk factor for colorectal cancer (CRC). The risk comes not only from known germline mutations but also from the other family-related mechanisms. Uncovering them would be an important step to improve the diagnosis and treatment of these patients.

**Method:**

Samples from 168 patients with advanced CRC were collected and applied to next-generation sequencing of 624 pan-cancer genes. Genomic mutations and significantly mutated genes were identified. Significantly mutated genes and co-mutated genes were used to cluster patients. For each cluster of patients, mutational signatures were extracted. The identified mutational signatures were further validated in the other independent cohort.

**Result:**

Significantly mutated genes including *TP53*, *APC*, *KRAS*, and *SMAD4* were found associated with tumor mutational burden and microsatellite instability. *LRP1*, *ACVR2A*, and *SETBP1* were found co-mutated. Patients with mutations in *LRP1*, *ACVR2A*, and *SETBP1* tend to have a family history of cancer. Those patients tended to have right-sided tumors with high tumor mutational burden and microsatellite instability. Among them, signature analysis identified two possible etiologies, SBS10a (defective polymerase epsilon exonuclease domain) and SBS6 (defective DNA mismatch repair and microsatellite unstable tumors). These signatures were also found in another independent cohort.

**Conclusion:**

The gene cluster (*LRP1*, *ACVR2A*, and *SETBP1*) could be a good biomarker of these patients with a family risk, which was characterized by right-sidedness, high tumor mutational burden, and high microsatellite instability.

## Introduction

Colorectal cancer (CRC) is the third most prevalent malignancy in China (1). Its incidence increased 65.8% during 1990 - 2017 (2). Of CRC patients, those with a family history of cancer accounts for 16%~36% (3) from which only a small number of patients are caused by known mechanisms.

Currently, the treatment of CRC patients is still a challenge. The 5-year overall survival rate of CRC patients ranges from 52.4% to 68% (4, 5). For the patients with metastatic tumors, the 5-year overall survival rate sharply reduces to 12% (6). To improve the treatment efficacy, subtyping CRC patients with molecular biomarkers is a promising way. The well-known molecular biomarkers include MSI (microsatellite instability) and *KRAS*/*BRAF* status. MSI is a biomarker for immunotherapy (7) and *KRAS*/*BRAF* is for anti-epidermal growth factor receptor therapies (8). Besides, there are biomarkers which could be used to reduce toxicity. For example, *UGT1A1* is a negative biomarker of fluorouracil and irinotecan combination therapy (9). During treatment, CRC patients with *UGT1A1* heterogeneity have a higher rate of severe toxicity. These examples demonstrate the importance of novel molecular biomarkers in clinical practice.

With the accumulation of clinical data, the heterogeneity defined by one driver gene among patients becomes recognized in many cancers (10). For example, *KRAS*-mutant lung adenocarcinoma could be divided into three subtypes according to different combinations of co-mutations, which are *TP53* co-mutations (KP subtype), *LKB1* co-mutations (KL subtype), and *CDKN2A*/*CDKN2B* co-mutations (KC subtype) (11). These subtypes have significantly different clinical behaviors: the KP subtype has low latency (12) and a higher metastatic proclivity; the KL subtype is more aggressive than the KP subtype and selectively sensitive to the treatment of deoxycytidine analogs, for example, gemcitabine; KC subtype is characterized by lacking *NKX2-1* expression and poor prognosis. Thus, a combination of two or more genes as a biomarker could improve the accuracy of subtyping cancers.

This study aims to identify familial CRC with co-mutations and mutually exclusive mutations. First, we sequenced the primary tumor tissues from 168 CRC patients using a 624 pan-cancer gene panel. Genomic mutations were then called. Co-mutation and mutual exclusivity in all patients with those genomic mutations were analyzed. The identified co-mutated gene clusters were used to subtype patients. Association study was performed between clinical characteristics and the identified subtypes. The clinically significant subtype was further validated in another independent cohort.

## Method

### Patients and Sample Collection

This study received the approval of the Research Ethics Committee of First Hospital of Shanxi Medical University. It enrolled 255 patients diagnosed with primary CRC. Written informed consent from each patient was provided. Samples were collected by surgery or ultrasound-guided fine-needle aspiration. A total of 168 high-quality samples from primary CRC tumors were obtained. These samples were then fixed with 4% formalin buffer and embedded in paraffin wax within 24 hours. We also downloaded the COAD dataset (571 patients) from the TCGA data portal (https://tcga-data.nci.nih.gov/docs/publications/tcga/), from which 265 patients of the white race were included in the further analysis.

### DNA Sequencing and Genomic Analysis

The library construction, sequencing, and mutation calling were performed by the Clinical Laboratory Improvement Amendments (CLIA)/College of American Pathologists (CAP)-compliant Molecular Diagnostics Service Laboratory of Shanghai OrigiMed Co., Ltd. Briefly, library construction from the FFPE (Formalin-fixed, paraffin-embedded) tissues, sequencing, mapping, mutation calling, and annotation followed the previous studies (13, 14). FFPE tissues were first sliced into 4µm sections. One section was stained with hematoxylin and eosin and underwent a pathologist review. FFPE samples were qualified if the stained slice had at least 1cm^2^ area, 20% nucleated cellularity, and 20% tumor content. DNA was extracted from 10 unstained FFPE sections. A panel of 624 pan-cancer genes (**Supplemental Table 1**) was amplified. After amplification, DNA was quantified by the Qubit system (Invitrogen Corporation). DNA mass less than 500ng was excluded for further sequencing. Sequencing was performed on Illumina Novaseq 6000 sequencer (Illumina, San Diego, CA). Genomic mutations including short nucleotide (single nucleotide and small insert and deletion), copy number variation, and fusion were called. Tumor mutational burden (TMB) was defined as the number of mutations per one million nucleotides. TMBs were split by 10 into low and high. MSI was called by the python package “MANTIS” with default parameters.

In mutational signature analysis, gene mutations in each sample were classified into 96 trinucleotide patterns according to the neighbor nucleotide context of mutations with the R package “MutationalPatterns” (15). Subsequently, the resulting mutational pattern matrix (96 trinucleotide patterns by 25 samples) was decomposed with non-negative matrix factorization. The optimum number of components of non-negative matrix factorization was determined by maximizing the logarithm likelihood.

### Functional Analysis of Genes

Gene ontology, KEGG (Kyoto Encyclopedia of Genes and Genomes) pathway, and GSEA (Gene Set Enrichment Analysis) were used to identify the functional enrichment of gene lists. Enrichment analysis of gene ontology and KEGG pathways was performed with the clusterProfiler package on the R platform (16). GSEA desktop application was downloaded from www.broadinstitute.org/gsea/index.jsp. Hallmark gene sets were used in the GSEA enrichment analysis. Gene ontology annotations, KEGG pathways, and GSEA hallmark gene sets (v7.3) were downloaded on March 31, 2021.

### Significantly Mutated Genes and Nucleotides

MutSigCV (17) was used to assess the significance of short nucleotide mutations (single nucleotide variant, small insert, and deletion mutations) according to its manual. MutSigCV measured the significance of non-silent somatic mutations by considering background patient-specific mutation frequency, cancer-specific mutational spectrum, and region-specific mutation heterogeneity.

To cluster gene mutation appearance in patients, we constructed a similarity matrix of genes versus genes. First, the appearance of gene mutations was compared between each pair of genes with Fisher’s exact test. Then the *P*-values were transformed with -log10(*P*-value) and assigned positive and negative signs for co-mutations and exclusive mutations, respectively. The transformed *P*-values were used as the co-mutation score between two genes. The association score between each gene and clinical characteristics was also calculated with -log10(*P*-value).

Signature analysis of DNA mutations was used to disclose the possible mutation mechanisms. The analysis procedure followed the manual of the MutationalPatterns package. First, mutational signatures were extracted with the vb_factorize function of the ccfindR package using the Bayesian algorithm and gamma priors. These extracted signatures were compared to the COSMIC signatures (V3.1) (18) with the rename_nmf_signatures function of the MutationalPatterns package. Cosine similarity > 0.85 to an existing COSMIC signature was defined as a significant association.

### Statistical Analysis

Mutation frequency comparison was performed with Fisher’s exact test. The *P*-values from Fisher’s exact test were adjusted with the Benjamin-Hochberg method. The adjusted *P*-values less than 0.05 were defined as statistically significant.

## Result

### Patient Characteristics

**Figure 1:**
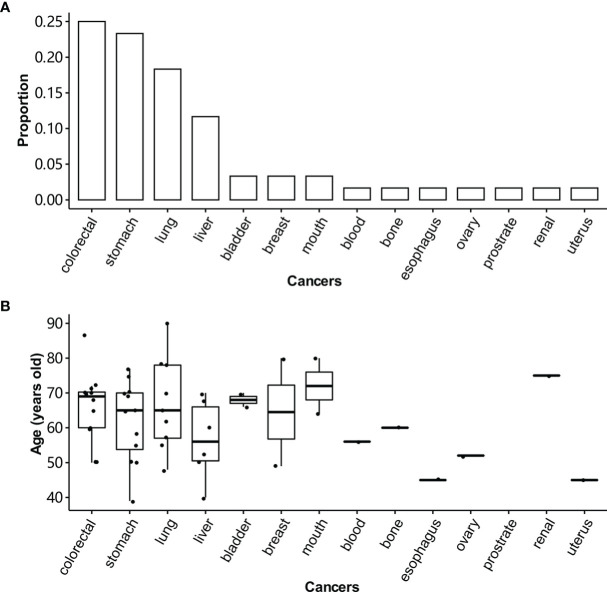
Distribution of cancers in the family members of CRC patients. **(A)** The cancer frequency distribution of family members for CRC patients. Patients with a family history are the ones whose parents, siblings, or children have cancer. **(B)** The age distribution of family members over tissue for CRC patients.

A total of 255 patients with CRC were enrolled in this study. Of them, 168 patients had enough qualified samples for sequencing and were included in the following analysis (**Table 1**). The percentages of patients at stage III or IV were 58.3% and 41.7%, respectively. The percentage of male patients was higher than that of females (55.7% vs. 44.3%). The age of patients ranged from 17 to 81 years, with a median of 60 years. Patients with the age < 60 and ≥ 60 were defined as young and old patients, respectively. High TMB accounted for 5.5% of patients. And 5.9% of patients were of MSI. Patients with a family history were defined as the ones whose parents, siblings, or children have cancer. A family history of colorectal, stomach, lung, and liver cancers was the most frequent phenotype for the patients (**Figure 1A**). They were most diagnosed with cancers between 65 and 75 years old (**Figure 1B**). The incidence of lung cancer in the family members was significantly higher than that of breast, bladder, and mouth cancers.

**Table 1:** Patient characteristics.

	Overall (N=168)
Age	
Young	64 (38.1%)
Old	104 (61.9%)
Gender	
Female	72 (42.9%)
Male	96 (57.1%)
Stage	
III	98 (58.3%)
IV	70 (41.7%)
Grade	
Low	94 (56.0%)
High	37 (22.0%)
Missing	37 (22.0%)
Sidedness	
Left	115 (68.5%)
Right	34 (20.2%)
Missing	19 (11.3%)
TMB	
Low	147 (87.5%)
High	21 (12.5%)
MSI	
MSS	155 (92.3%)
MSI-H	11 (6.5%)
Missing	2 (1.2%)
Family History	
No	101 (60.1%)
Yes	47 (28.0%)
Missing	20 (11.9%)

### Genomic Mutation Landscape

**Figure 2:**
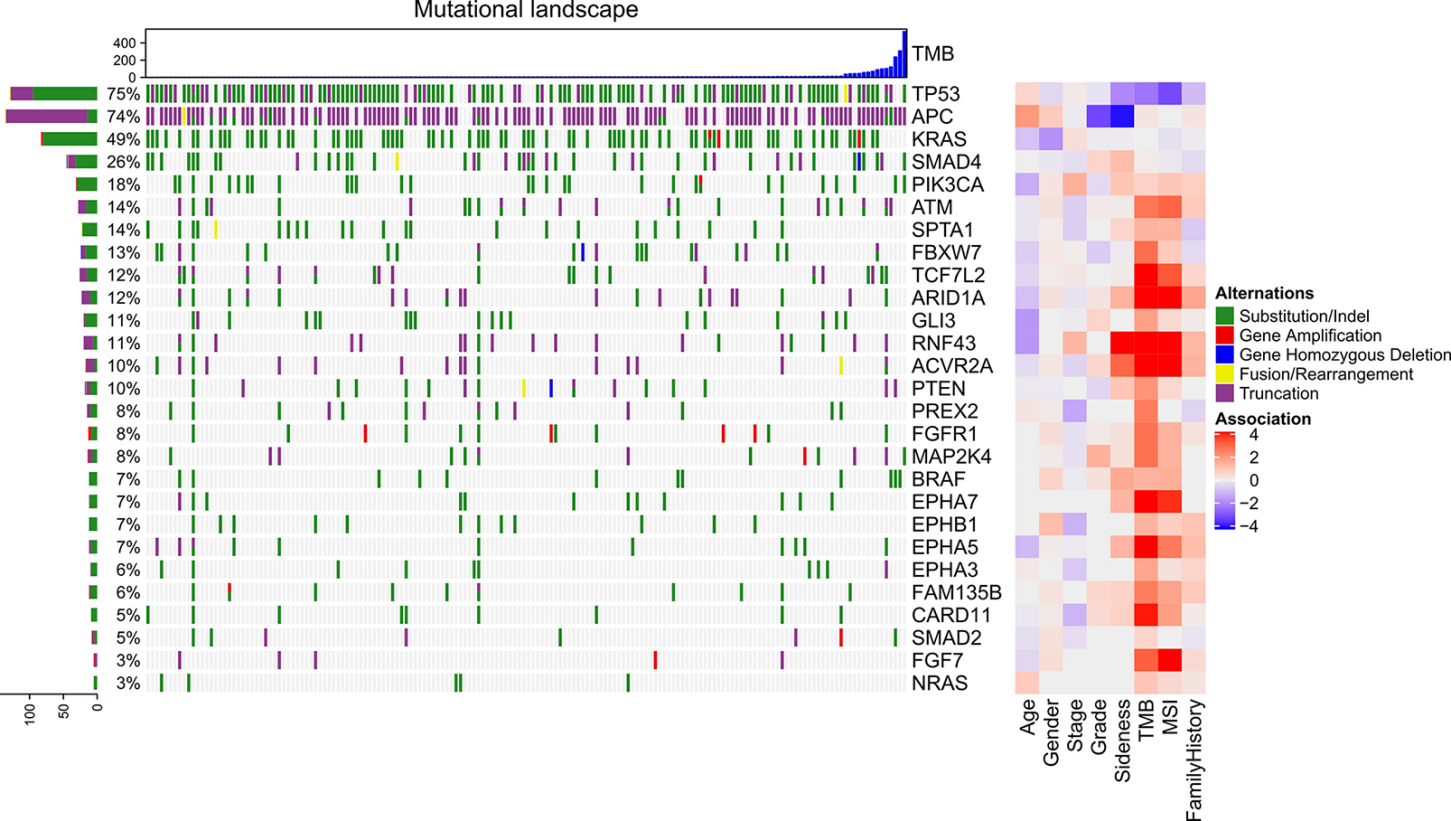
Genomic mutations in advance CRC and their association with clinical characteristics. The top bar plot indicates the tumor mutational burden of each sample. The left bar plot indicates the frequency sum of mutations in each gene. The middle heatmap indicates the mutations of each gene in each sample. The right heatmap indicates the association between each gene and clinical character.

The genomic mutations were shown in **Figure 2**. The top-5 mutated genes were *TP53*, *APC*, *KRAS*, *SMAD4*, and *PIK3CA*. After removing the mutational bias in specific genes, genome regions, and patients, MutSigCV identified 27 significantly mutated genes. These genes included *TP53*, *SMAD4*, *FBXW7*, *PIK3CA*, and *APC* (**Supplemental Table 2**). In the first column (**Figure 2**), there were multiple mutations in *APC* and *TP53*, which led to a total frequency sum larger than 100%. These significant genes were compared to clinical characteristics including age, gender, stage, grade, sidedness, TMB, MSI, and family history with Fisher’s exact test. The right column of the heatmap indicated the association score between each gene and clinical characteristics (**Figure 2**). *APC* was specifically mutated in the left-sided (descendent colon, sigmoid colon, and rectum) CRC while *RNF43* in the right-sided (cecum, ascendant colon, and transverse colon) CRC. Patients with *TP53* mutations tended to be microsatellite-stable. *ARID1A*, *RNF43*, *ACVR2A*, and *EPHA7* were positively correlated with TMB and MSI. The association between family history cancers and gene mutations were shown in **Supplemental Figure 1**.

### Co-Mutation Analysis

**Figure 3:**
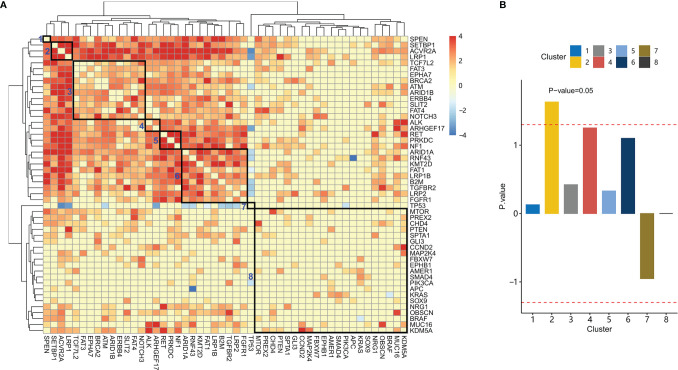
Co-mutation between genes and their association with clinical characteristics. **(A)** Hierarchical clustering of the co-mutation score between genes. Eight gene clusters were enclosed with rectangles. From left to right, these clusters were labeled 1-8. **(B)** Association scores for each cluster were plotted. The dashed red line indicates an association score at *P*-value=0.05.

Genes were hierarchically clustered based on co-mutation scores with Euclidian distance and Ward agglomeration method (**Figure 3A**). Eight clusters were identified.

We further studied the association between these gene clusters and family history. For each gene cluster, we analyzed the association between a family history of cancer and gene mutations in the gene cluster. Patients with mutations in gene cluster 2 have a significantly higher proportion of patients with a family history of cancer than those without mutations in gene cluster 2 (**Figure 3B**, *P*-value= 0.02). These patients were denoted as LAS subtype, which had a mutation in *LRP1*, *ACVR2A*, or *SETBP1* (LAS).

### Characteristics of LAS Subtype Patients

**Figure 4:**
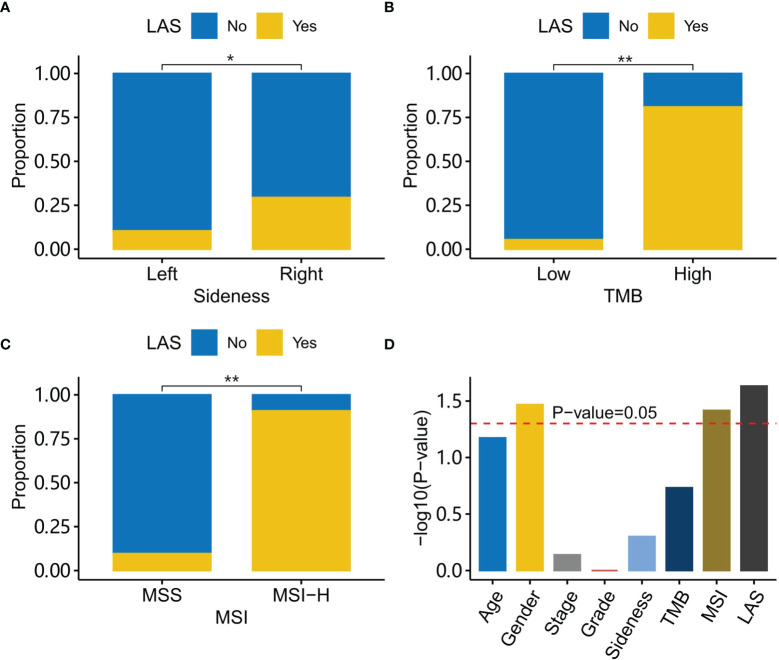
Characteristics of LAS subtype patients. **(A)** LAS subtype patients have a higher percentage of right-side tumors than the left-side tumor. **(B)** LAS subtype patients have a higher percentage of high TMB tumors than low TMB tumors. **(C)** LAS subtype patients have a higher percentage of MSI-H tumors than MSS tumors. **(D)** Association with a family history of cancer for each clinical character is plotted. The red dash line indicates the association score at *P*-value=0.05. “*” and “**” denote *P*-value < 0.05 and *P*-value < 0.001, respectively.

Clinical characteristics including gender, age, stage, sidedness, and tumor grade were compared between LAS subtype patients and the other patients. It was found that LAS subtype patients were significantly different from the other patients in sidedness, TMB, and MSI (**Figures 4A–C**). LAS subtype patients tended to have right-sided CRC (Fisher’s exact test, *P*-value=1.14e-2), high MSI (Fisher’s exact test, *P*-value=1.25e-8), and high TMB (Fisher’s exact test, *P*-value= 6.25e-9). And the family history was significantly associated with gender, MSI, and LAS subtype (**Figure 4D**).

KEGG pathway enrichment analysis indicated that mutations in pathways including mismatch repair, cholesterol metabolism, and cGMP-PKG signaling pathways were significantly present in LAS subtype patients (*P*-value < 0.001) (**Supplemental Figure 2**).

### Signature Analysis of LAS Subtype Patients

**Figure 5:**
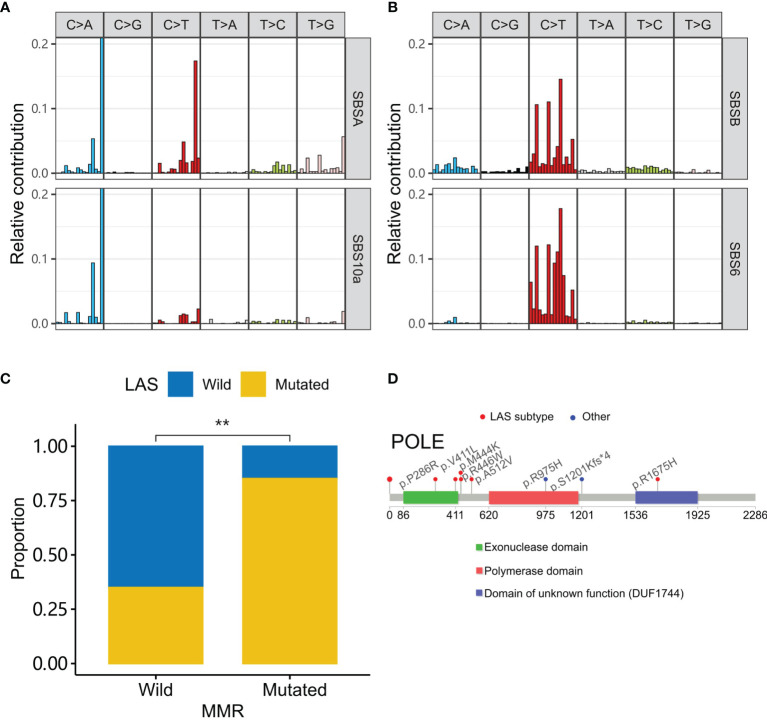
LAS subtype-associated mutation signatures. **(A, B)** Two signatures (SBSA and SBSB) are extracted in the cohort. They are respectively similar to the signatures, SBS10a and SBS6 of the COSMIC database. **(C)** MMR gene (*MSH2*, *MSH3*, *MSH6*, *MLH1*, *MLH3*, *PMS2*, *PMS1*, *EXO1*, *POLD3*, *PCNA*, *RPA1*, *HMGB1*, *RFC1*, and *LIG1*) mutations are higher in the LAS subtype CRC. **(D)** Mutations in the tumors of LAS subtype patients are around the exonuclease domain of polymerase epsilon. “**” denote *P*-value < 0.001.

We further identified the potential pathogenic mechanism in the LAS patients with mutational signature analysis (15). Two mutational signatures (SBSA and SBSB) were extracted. Then they were compared to the COSMIC signature database with cosine similarity (17). SBSA and SBSB were found similar to SBS10a (cosine similarity=0.83) and SBS6 (cosine similarity=0.91), respectively (**Figures 5A, B**). The etiology of SBS10a is possibly driven by the *POLE* (polymerase epsilon) exonuclease domain mutations. And the SBS6 signature could be caused by defective DNA MMR (mismatch repair) and associated with MSI. In contrast, patients without LAS mutations had a signature similar to SBS1 (cosine similarity=0.879, **Supplemental Figure 3**). The etiology of SBS1 is spontaneous deamination of 5-methylcytosine.

To test whether SBSA and SBSB were involved in the related defection, we compared the tumor gene mutations of LAS subtype patients to that of the other patients in MMR genes (19) and *POLE* (**Figures 5C, D**). MMR genes (*MSH2*, *MSH3*, *MSH6*, *MLH1*, *MLH3*, *PMS2*, *PMS1*, *EXO1*, *POLD3*, *PCNA*, *RPA1*, *HMGB1*, *RFC1*, and *LIG1*) and *POLE* mutations were preferentially mutated in the tumors of the LAS subtype patients than the others. And most of *POLE* mutations were present in or around the *POLE* exonuclease domain.

### LAS Subtype Patients in the Other Independent Cohorts

**Figure 6:**
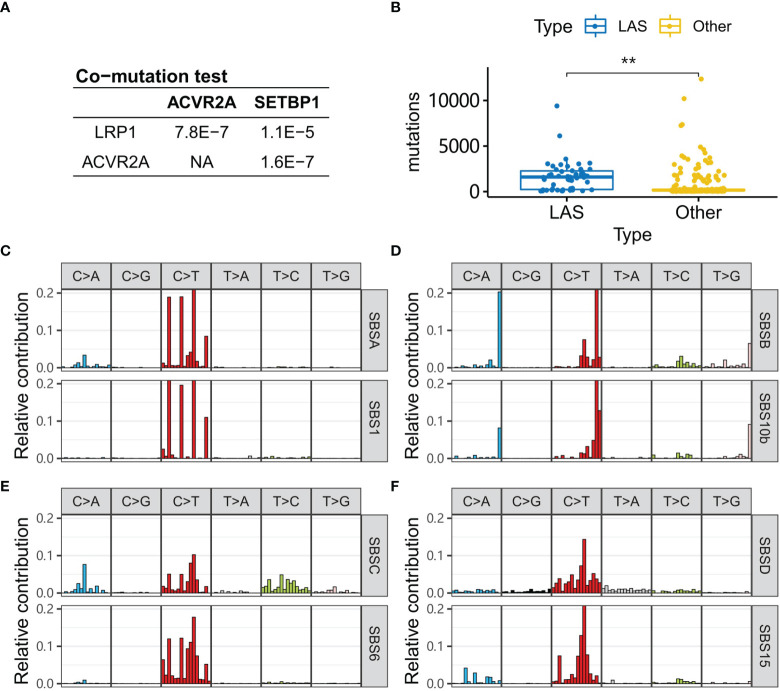
LAS type patients in the COAD cohort. **(A)** *P*-values of the co-mutation between each pair of *LRP1*, *ACVR2A*, and *SETBP1* are calculated with Fisher’s exact test. **(B)** Tumors of LAS subtype patients have more mutations than the other patients. **(C–F)** Four signatures (SBSA, SBSB, SBSC, SBSD) are extracted from the COAD cohort. They are respectively similar to the signatures, SBS1, SBS10b, SBS6, and SBS15, in the COSMIC database. “**” denote *P*-value < 0.001.

To validate the co-mutation of LAS genes, an independent cohort (COAD dataset) was used. In the COAD dataset, patients of the white race were selected. Consistent with the results in the Chinese cohort, *LRP1* was significantly co-mutated with *SETBP1* and *ACVR2A* (Fisher’s exact test, *P*-value<1.1e-5, **Figure 6A**). And LAS subtype patients of the COAD cohort tended to have right-sided CRC (Fisher’s exact test, *P*-value=8.13e-15, **Supplemental Figure 4**) and a significantly higher mutation number (Wilcox’s rank-sum test, *P*-value=1.4e-13, **Figure 6B**). With mutational signature analysis, four mutational signatures were extracted (**Figures 6C–F**). They were similar to SBS1 (spontaneous or enzymatic deamination of 5-methylcytosine), SBS10b (defective DNA mismatch repair), SBS6 (defective DNA mismatch repair), and SBS15 (defective DNA mismatch repair) of COSMIC signatures (**Figure 5C**). And the corresponding cosine similarity was 0.91, 0.86, 0.78, and 0.87.

## Discussion

In this work, we firstly studied the characteristics of CRC patients with a family history of cancer. A total of 255 Chinese CRC patients were enrolled. The most diagnosed cancers for their first-degree family members were colorectal, stomach, lung, and liver cancers. Of them, lung cancer was not included in the Bethesda and Amsterdam diagnostic criteria for Lynch syndrome. However, the incidence of lung cancer in the family members of CRC patients was significantly higher than the other cancers including bladder, breast, and mouth cancers. In spite, they were not mentioned in the Bethesda and Amsterdam diagnostic criteria. The high incidence of lung cancer in the Chinese population could partly explain the result, but it could not explain why lung cancer was significantly (3 times) higher than breast cancer in the family members of CRC patients since the incidence of lung and breast cancer were comparable in the Chinese population (1). The above result suggested that lung cancer diagnosed in a family member might be a risk of CRC in the Chinese population.

To subtype the CRC patients with a family history of cancer with genomic mutations, we sequenced 168 CRC high-quality samples. Significantly mutated genes were called with MutSigCV and compared between patient groups with different clinical characteristics. *APC* and *RNF43* were respectively highly mutated in patients with left-side and right-side CRC, which was consistent with previous studies (20, 21). Besides, *ARID1A*, *RNF43*, *ACVR2A*, and *EPHA7* mutations were found significantly associated with both TMB and MSI. These results had also been observed in several studies. For example, *ARID1A* mutations were reported to be associated with higher TMB and MSI in a large European study (22). *RNF43* mutations were significantly associated with MSI (21). *ACVR2A* hyper-mutation in the tumor of patients with MSI was reported in Chinese patients with gastric cancers (23). But we failed to find any evidence about the *ACVR2A* mutational preference in the tumors of CRC patients with MSI.

Next, genes were clustered by co-mutation scores. Eight gene clusters were obtained. Using the eight gene clusters, patients with the mutations in each gene cluster were labeled as eight subtypes accordingly. Of the eight subtypes, one subtype named LAS was significantly associated with family history. Patients of the LAS subtype had mutations in the *LRP1* (low-density lipoprotein receptor-related protein-1), *ACVR2A* (activin A Receptor Type 2A), and *SETBP1* (SET Binding Protein 1). Of the three genes, *LRP1* was studied in breast cancers (24) but rarely in CRC. *LRP1* functions to mediate the clearance of various molecules from the extracellular matrix (25). *ACVR2A* is a member of the transforming growth factor-beta (TGF-β) family, involving the regulation of cell differentiation, migration, proliferation, and apoptosis of colon cancer (26, 27). The function of *SETBP1* was still unclear. It could interfere with the TGF-β signaling pathway (28). We found that LAS subtype patients had a higher rate of high TMB and MSI and more frequently appeared in right-sided CRC. As a matter of fact, low expression of *LRP1* in colon adenocarcinomas is associated with MSI (29). *ACVR2A* contains two DNA microsatellite sites in exon 3 and exon 10, which could be associated with MSI. No report is found on the association between *LRP1* mutations and MSI in CRC.

We were also interested in the characteristics associated with a family history of cancer. Sidedness, TMB, MSI, and LAS subtype were compared between patients with and without a family history of cancer. The results showed that the LAS subtype was significantly associated with a family history of cancer. LAS subtype could be a better indicator of a family history of cancer. Nevertheless, the mechanism behind the association of the LAS subtype and a family history of cancer was still unclear. Mutational signature analysis shed some light on the puzzle. Using Bayesian non-negative matrix decomposition, two mutational signatures were extracted. They both had a cosine similarity > 0.8 to one of the COSMIC signatures. The two COSMIC signatures were SBS6 (defective polymerase epsilon exonuclease domain) and SBS10a (defective DNA mismatch repair and microsatellite unstable tumors). Further checking the mutations in the polymerase epsilon exonuclease domain validated the hypothesis that LAS subtype patients had more mutations in the polymerase epsilon exonuclease domain. The mutations in the mismatch repair genes were also enriched in the LAS subtype patients. LAS subtype could be the combinatory results of the two mechanisms.

Another interesting question is whether the LAS subtype exists in the white race patients? The results in the COAD cohort validated the hypothesis that the LAS genes (*LRP1*, *ACVR2A*, and *SETBP1*) were significantly co-mutated in the COAD cohort. The characteristics of LAS subtype patients including right-sidedness, MSI, TMB, and *TP53* mutations were consistent with that in the Chinese population. Etiology analysis of the genomic mutation in CRC identified four signatures. Of them, one signature similar to SBS6 in the COSMIC signature database also existed in the Chinese population and the other three signatures (SBS6, SBS10b, and SBS15) were similar to mismatch repair signatures, like the counterpart, SBS10a, in the Chinese population. Besides, one specific mutational signature was found, which was related to the deamination of 5-methylcytosine.

LAS subtyping could have multiple applications. Considering the high TMB and MSI characteristics of LAS subtype patients, we hypothesize that patients of the LAS subtype could be treated with immunotherapy (30). Family members of LAS subtype patients were prone to lung cancer, which could be used in the prediction of lung cancer in their family members. Besides, LAS subtyping could help distinguish primary right-sided tumors from primary left-sided tumors, especially when tumors appear on both sides.

## Conclusion

This work identified a cluster of co-mutation genes (*LRP1*, *ACVR2A*, and *SETBP1*) and found these genes were significantly associated with a family history of cancer. Patients with these gene mutations tended to have right-sided, high TMB, and MSI tumors. And this subtype exists in both the white and Chinese races.

## Data Availability Statement

The datasets presented in this article are not readily available because of patient privacy concerns. Requests to access the datasets should be directed to Yi Ba, bayi@tjmuch.com.

## Ethics Statement

The studies involving human participants were reviewed and approved by the Research Ethics Committee of First Hospital of Shanxi Medical University. The patients/participants provided their written informed consent to participate in this study.

## Author Contributions

HH and YB conceived and designed this study. HH, TD, YG, HC, XC, JD, YY, and ZG collected data and performed the analysis. HH, TD, and YB wrote the manuscript. All authors contributed to the article and approved the submitted version.

## Funding

This work was supported by grants from the National Natural Science Foundation of China (Nos. 82072664, 81772629, 81974374, 82173125, 81802363, 81702431), Tianjin Science Foundation (Nos. 18JCQNJC81900, 18JCYBJC92000, 18JCYBJC25400, 18JCYBJC92900) and the Science & Technology Development Fund of the Tianjin Education Commission for Higher Education (2018KJ046, 2017KJ227). The funders had no role in the study design, the data collection and analysis, the interpretation of the data, the writing of the report, and the decision to submit this article for publication.

## Conflict of Interest

The authors declare that the research was conducted in the absence of any commercial or financial relationships that could be construed as a potential conflict of interest.

## Publisher’s Note

All claims expressed in this article are solely those of the authors and do not necessarily represent those of their affiliated organizations, or those of the publisher, the editors and the reviewers. Any product that may be evaluated in this article, or claim that may be made by its manufacturer, is not guaranteed or endorsed by the publisher.

## References

[1] Sun D Cao M Li H He S Chen W. Cancer Burden and Trends in China: A Review and Comparison With Japan and South Korea. Chin J Cancer Res (2020) 32(2):129–39. doi: 10.21147/j.issn.1000-9604.2020.02.01 PMC7219092 32410791

[2] Yin J Bai Z Zhang J Zheng Z Yao H Ye P. Burden of Colorectal Cancer in China, 1990–2017: Findings From the Global Burden of Disease Study 2017. Chin J Cancer Res (2019) 31(3):489–98. doi: 10.21147/j.issn.1000-9604.2019.03.11 PMC6613508 31354218

[3] Clarke CN Scott Kopetz E. Molecular Markers and Mutational Analysis. In: Chang GJ, editor. Rectal Cancer: Modern Approaches to Treatment [Internet]. Cham: Springer International Publishing (2018). p. 295–312. doi: 10.1007/978-3-319-16384-0_19

[4] Yuan Y Li M-D Hu H-G Dong C-X Chen J-Q Li X-F. Prognostic and Survival Analysis of 837 Chinese Colorectal Cancer Patients. World J Gastroenterol (2013) 19(17):2650–9. doi: 10.3748/wjg.v19.i17.2650 PMC3645383 23674872

[5] Li X Zhou Y Luo Z Gu Y Chen Y Yang C. The Impact of Screening on the Survival of Colorectal Cancer in Shanghai, China: A Population Based Study. BMC Public Health (2019) 19:1016. doi: 10.1186/s12889-019-7318-8 31357981 PMC6664771

[6] Rawla P Sunkara T Barsouk A. Epidemiology of Colorectal Cancer: Incidence, Mortality, Survival, and Risk Factors. Prz Gastroenterol (2019) 14(2):89–103. doi: 10.5114/pg.2018.81072 31616522 PMC6791134

[7] Overman MJ McDermott R Leach JL Lonardi S Lenz HJ Morse MA. Nivolumab in Patients With Metastatic DNA Mismatch Repair-Deficient or Microsatellite Instability-High Colorectal Cancer (CheckMate 142): An Open-Label, Multicentre, Phase 2 Study. Lancet Oncol (2017) 18(9):1182–91. doi: 10.1016/S1470-2045(17)30422-9 PMC6207072 28734759

[8] Van Cutsem E Köhne C-H Láng I Folprecht G Nowacki MP Cascinu S. Cetuximab Plus Irinotecan, Fluorouracil, and Leucovorin as First-Line Treatment for Metastatic Colorectal Cancer: Updated Analysis of Overall Survival According to Tumor KRAS and BRAF Mutation Status. J Clin Oncol (2011) 29(15):2011–9. doi: 10.1200/JCO.2010.33.5091 21502544

[9] Gao J Zhou J Li Y Lu M Jia R Shen L. UGT1A1*6/*28 Polymorphisms Could Predict Irinotecan-Induced Severe Neutropenia Not Diarrhea in Chinese Colorectal Cancer Patients. Med Oncol (2013) 30(3):604. doi: 10.1007/s12032-013-0604-x 23686699

[10] Scheffler M Ihle MA Hein R Merkelbach-Bruse S Scheel AH Siemanowski J. K-Ras Mutation Subtypes in NSCLC and Associated Co-Occuring Mutations in Other Oncogenic Pathways. J Thorac Oncol (2019) 14(4):606–16. doi: 10.1016/j.jtho.2018.12.013 30605727

[11] Skoulidis F Heymach JV. Co-Occurring Genomic Alterations in non-Small Cell Lung Cancer Biology and Therapy. Nat Rev Cancer (2019) 19(9):495–509. doi: 10.1038/s41568-019-0179-8 31406302 PMC7043073

[12] Jamal-Hanjani M Wilson GA McGranahan N Birkbak NJ Watkins TBK Veeriah S. Tracking the Evolution of Non-Small-Cell Lung Cancer. N Engl J Med (2017) 376(22):2109–21. doi: 10.1056/NEJMoa1616288 28445112

[13] Chen X Bu Q Yan X Li Y Yu Q Zheng H. Genomic Mutations of Primary and Metastatic Lung Adenocarcinoma in Chinese Patients. J Oncol (2020) 2020:e6615575. doi: 10.1155/2020/6615575 PMC7787720 33488709

[14] Xu S Guo Y Zeng Y Song Z Zhu X Fan N. Clinically Significant Genomic Alterations in the Chinese and Western Patients With Intrahepatic Cholangiocarcinoma. BMC Cancer (2021) 21(1):152. doi: 10.1186/s12885-021-07792-x 33579226 PMC7879680

[15] Blokzijl F Janssen R van Boxtel R Cuppen E. MutationalPatterns: Comprehensive Genome-Wide Analysis of Mutational Processes. Genome Med (2018) 10(1):33. doi: 10.1186/s13073-018-0539-0 29695279 PMC5922316

[16] Yu G Wang L-G Han Y He Q-Y. Clusterprofiler: An R Package for Comparing Biological Themes Among Gene Clusters. OMICS (2012) 16(5):284–7. doi: 10.1089/omi.2011.0118 PMC3339379 22455463

[17] Lawrence MS Stojanov P Polak P Kryukov GV Cibulskis K Sivachenko A. Mutational Heterogeneity in Cancer and the Search for New Cancer-Associated Genes. Nature (2013) 499(7457):214–8. doi: 10.1038/nature12213 PMC3919509 23770567

[18] Alexandrov LB Kim J Haradhvala NJ Huang MN Tian Ng AW Wu Y. The Repertoire of Mutational Signatures in Human Cancer. Nature (2020) 578(7793):94–101. doi: 10.1038/s41586-020-1943-3 32025018 PMC7054213

[19] Li G-M. Mechanisms and Functions of DNA Mismatch Repair. Cell Res (2008) 18(1):85–98. doi: 10.1038/cr.2007.115 18157157

[20] Huang W Li H Shi X Lin M Liao C Zhang S. Characterization of Genomic Alterations in Chinese Colorectal Cancer Patients. Jpn J Clin Oncol (2021) 51(1):120–9. doi: 10.1093/jjco/hyaa182 33106877

[21] Matsumoto A Shimada Y Nakano M Oyanagi H Tajima Y Nakano M. RNF43 Mutation is Associated With Aggressive Tumor Biology Along With BRAF V600E Mutation in Right-Sided Colorectal Cancer. Oncol Rep (2020) 43(6):1853. doi: 10.3892/or.2020.7561 32236609 PMC7160543

[22] Tokunaga R Xiu J Goldberg RM Philip PA Seeber A Battaglin F. The Impact of ARID1A Mutation on Molecular Characteristics in Colorectal Cancer. Eur J Cancer (2020) 140:119–29. doi: 10.1016/j.ejca.2020.09.006 PMC8009046 33080474

[23] Zhao L Zhang J Qu X Yang Y Gong Z Yang Y. Microsatellite Instability-Related ACVR2A Mutations Partially Account for Decreased Lymph Node Metastasis in MSI-H Gastric Cancers. Onco Targets Ther (2020) 13:3809–21. doi: 10.2147/OTT.S247757 PMC7211323 32440149

[24] Berquand A Meunier M Thevenard-Devy J Ivaldi C Campion O Dedieu S. A Gentle Approach to Investigate the Influence of LRP-1 Silencing on the Migratory Behavior of Breast Cancer Cells by Atomic Force Microscopy and Dynamic Cell Studies. Nanomedicine (2019) 18:359–70. doi: 10.1016/j.nano.2018.10.012 30419363

[25] Le CC Bennasroune A Collin G Hachet C Lehrter V Rioult D. LRP-1 Promotes Colon Cancer Cell Proliferation in 3D Collagen Matrices by Mediating DDR1 Endocytosis. Front Cell Dev Biol (2020) 8. doi: 10.3389/fcell.2020.00412 PMC7283560 32582700

[26] Olsen OE Wader KF Hella H Mylin AK Turesson I Nesthus I. Activin A Inhibits BMP-Signaling by Binding ACVR2A and ACVR2B. Cell Commun Signal (2015) 13:27. doi: 10.1186/s12964-015-0104-z 26047946 PMC4467681

[27] Loomans HA Andl CD. Intertwining of Activin A and Tgfβ Signaling: Dual Roles in Cancer Progression and Cancer Cell Invasion. Cancers (Basel) (2014) 7(1):70–91. doi: 10.3390/cancers7010070 25560921 PMC4381251

[28] Coccaro N Tota G Zagaria A Anelli L Specchia G Albano F. SETBP1 Dysregulation in Congenital Disorders and Myeloid Neoplasms. Oncotarget (2017) 8(31):51920–35. doi: 10.18632/oncotarget.17231 PMC5584301 28881700

[29] Boulagnon-Rombi C Schneider C Leandri C Jeanne A Grybek V Bressenot AM. LRP1 Expression in Colon Cancer Predicts Clinical Outcome. Oncotarget (2018) 9(10):8849–69. doi: 10.18632/oncotarget.24225 PMC5823651 29507659

[30] Schrock AB Ouyang C Sandhu J Sokol E Jin D Ross JS. Tumor Mutational Burden is Predictive of Response to Immune Checkpoint Inhibitors in MSI-High Metastatic Colorectal Cancer. Ann Oncol (2019) 30:1096–103. doi: 10.1093/annonc/mdz134 31038663

