# Supplementary material for: Gene Mutational Clusters in the Tumors of Colorectal Cancer Patients With a Family History of Cancer
Source: Front Oncol. 2022 Jun 24;12:814397. doi: 10.3389/fonc.2022.814397 (PMC9266985; doi:10.3389/fonc.2022.814397)
Supplement: Supplementary file 1 [file DataSheet_1.docx]

Supplementary Material

# Supplementary Data

**Supplemental Figure 1.** **The genomic mutation associated with a family history of cancer.**

The association score between genes and a family history of cancers. A *P*-value below 0.05 is indicated with a dot in the cells.

**Supplemental Figure 2.** **Functional comparison between LAS subtype and other patients**

KEGG pathway enrichment is compared between LAS subtype patients and the other patients.

**Supplemental Figure 3. Non-LAS subtype-associated mutation signatures**

A signature (SBSA) is extracted from the Chinese cohort. It is similar to the SBS1 of the COSMIC signatures.

**Supplemental Figure 4. LAS subtype patients are prone to have right-sided tumors in the COAD cohort.**

# Supplementary Figures and Tables

## Supplementary Figures


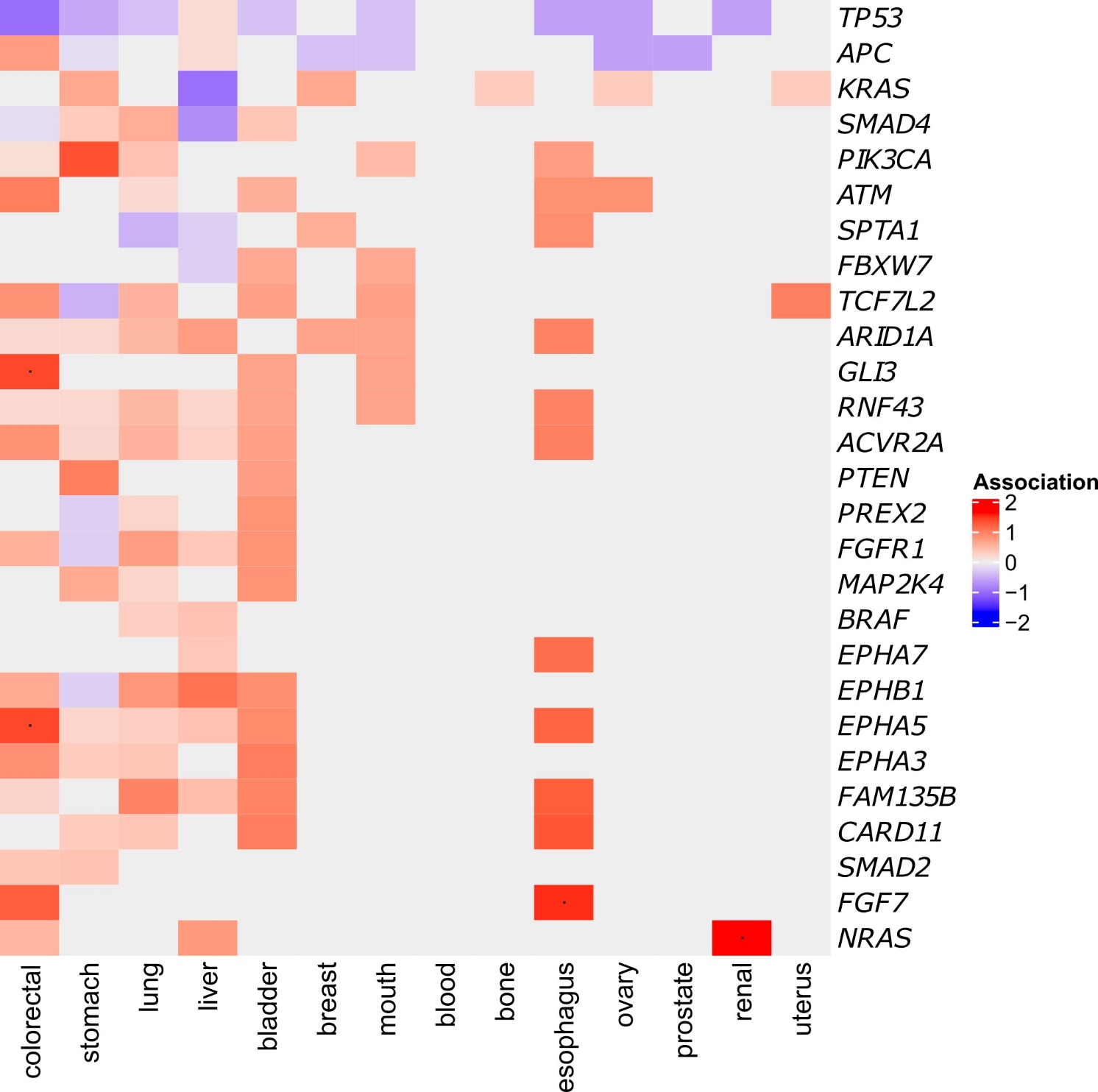


**Supplementary Figure 1.** **The genomic mutation associated with a family history of cancer.** The association score between genes and a family history of cancers. A *P*-value below 0.05 is indicated with a dot in the cells.


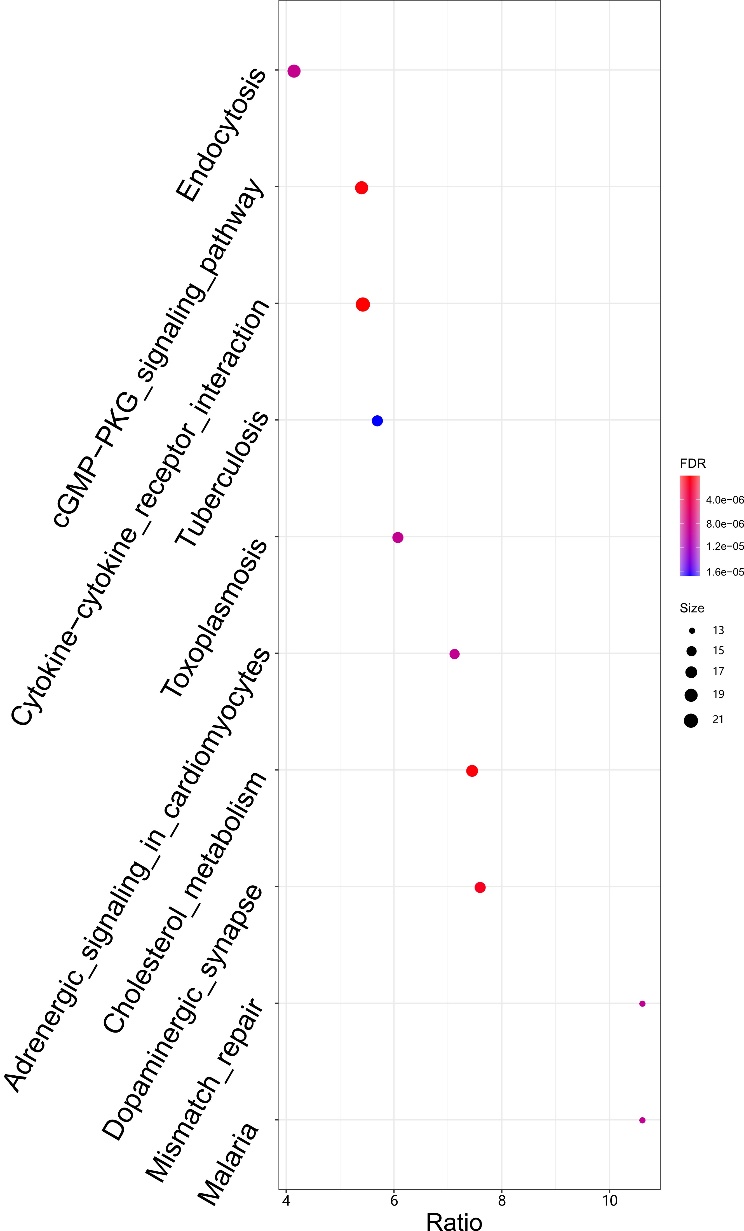


**Supplementary Figure 2. Functional comparison between LAS subtype and other patients.** KEGG pathway enrichment is compared between LAS subtype patients and the other patients.


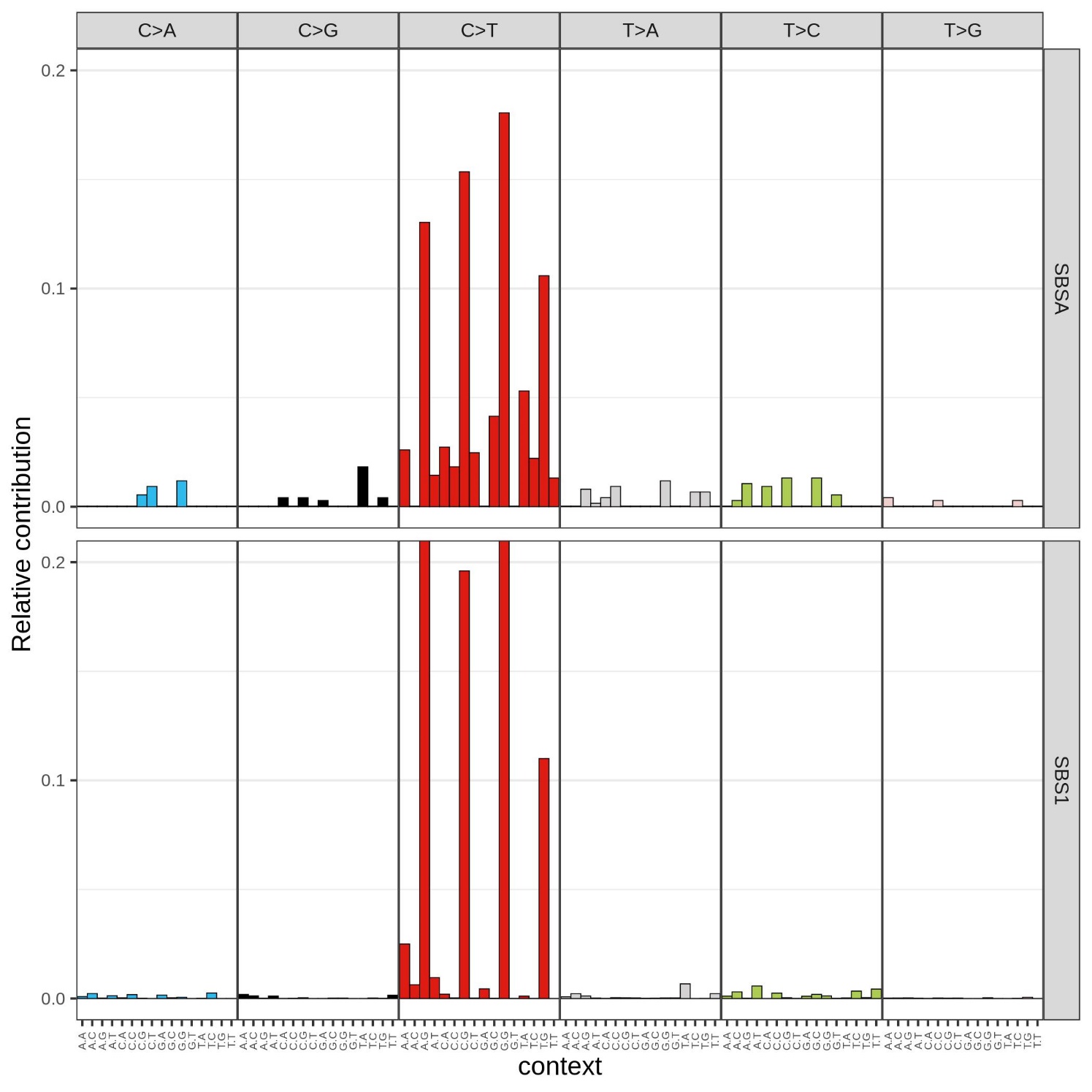


**Supplemental Figure 3. Non-LAS subtype-associated mutation signatures**

A signature (SBSA) is extracted from the Chinese cohort. It is similar to the SBS1 of the COSMIC signatures.


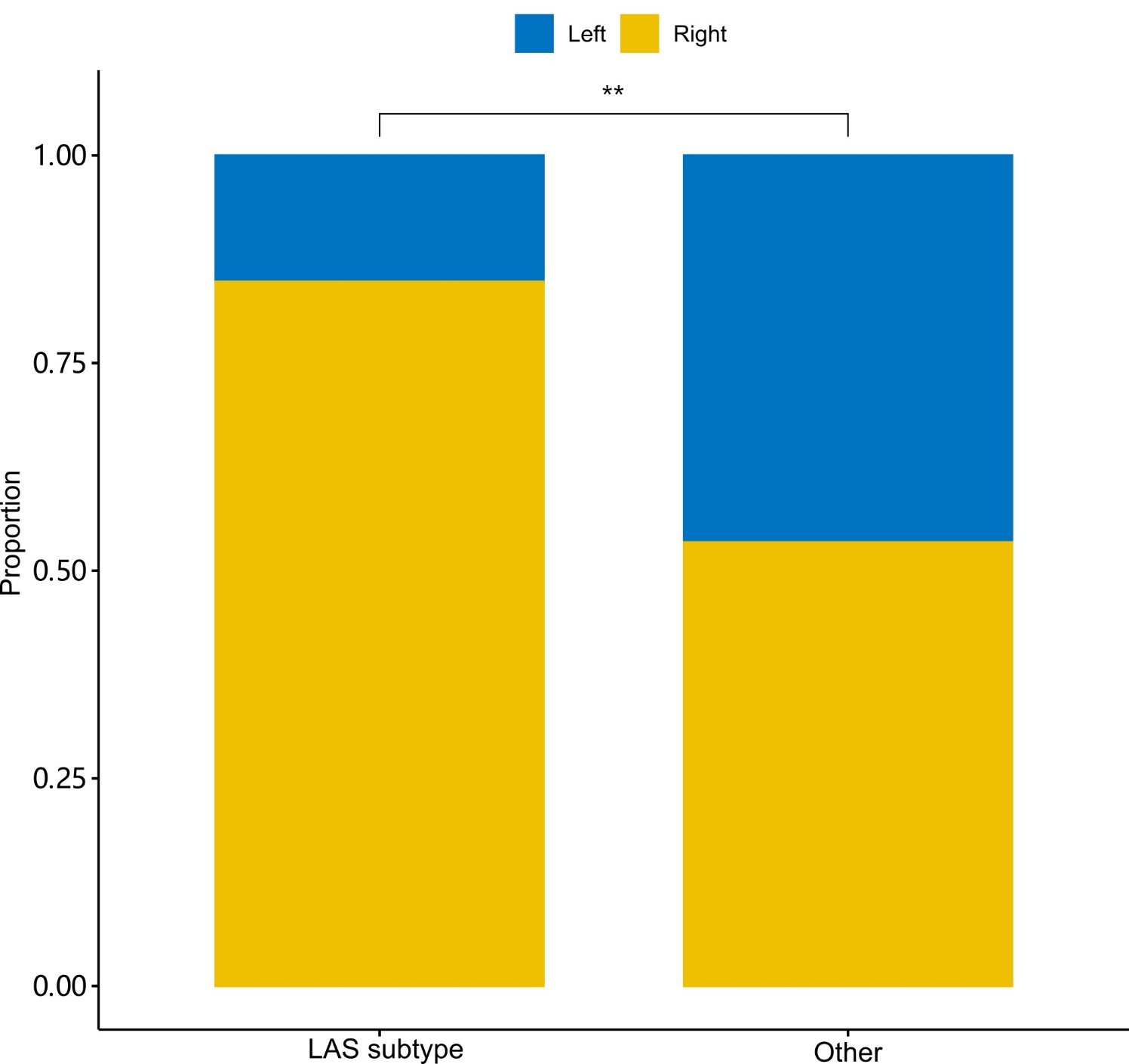


**Supplemental Figure 4. LAS subtype patients are prone to have right-sided tumors in the COAD cohort.**

## Supplementary Tables

**Supplemental Table 1. The targeted 624 pan-cancer genes**

| Gene names | | | | | | | |
| --- | --- | --- | --- | --- | --- | --- | --- |
| *ABCB1* | *ABL1* | *ABL2* | *ABRAXAS1* | *ACVR1* | *ACVR1B* | *ACVR2A* | *ADAM29* |
| *ADGRA2* | *AFF3* | *AKT1* | *AKT2* | *AKT3* | *ALK* | *AMER1* | *ANTXR2* |
| *APC* | *APEX1* | *APOBEC3B* | *AQP3* | *AR* | *ARAF* | *ARAP3* | *ARFRP1* |
| *ARHGAP4* | *ARHGAP6* | *ARHGDIA* | *ARHGEF10* | *ARHGEF25* | *ARHGEF3* | *ARID1A* | *ARID1B* |
| *ARID2* | *ASXL1* | *ATM* | *ATR* | *ATRX* | *AURKA* | *AURKB* | *AXIN1* |
| *AXIN2* | *AXL* | *B2M* | *BAP1* | *BARD1* | *BCAR4* | *BCL2* | *BCL2L1* |
| *BCL2L11* | *BCL2L2* | *BCL6* | *BCL7A* | *BCOR* | *BCORL1* | *BCR* | *BIRC3* |
| *BIRC5* | *BLK* | *BLM* | *BMPR1A* | *BMX* | *BRAF* | *BRCA1* | *BRCA2* |
| *BRD4* | *BRIP1* | *BTG1* | *BTG2* | *BTK* | *BUB1* | *CALR* | *CAMTA1* |
| *CARD11* | *CASP8* | *CBFB* | *CBL* | *CCN6* | *CCNB3* | *CCND1* | *CCND2* |
| *CCND3* | *CCNE1* | *CD1A* | *CD1B* | *CD1C* | *CD1D* | *CD1E* | *CD274* |
| *CD36* | *CD70* | *CD74* | *CD79A* | *CD79B* | *CDC42* | *CDC73* | *CDH1* |
| *CDK12* | *CDK2* | *CDK4* | *CDK6* | *CDK8* | *CDKN1A* | *CDKN1B* | *CDKN2A* |
| *CDKN2B* | *CEBPA* | *CFTR* | *CHD2* | *CHD4* | *CHEK1* | *CHEK2* | *CIC* |
| *CLDN18* | *CNOT2* | *COL1A1* | *CRBN* | *CREB3L1* | *CREB3L2* | *CREBBP* | *CRKL* |
| *CRLF2* | *CSF1R* | *CSF3R* | *CSK* | *CSNK1A1* | *CTCF* | *CTNNA1* | *CTNNA2* |
| *CTNNB1* | *CUL3* | *CUL4A* | *CXCR4* | *CYLD* | *CYP17A1* | *CYP2D6* | *DAXX* |
| *DCTN1* | *DDR1* | *DDR2* | *DEF6* | *DEK* | *DGCR8* | *DICER1* | *DIS3* |
| *DLC1* | *DNMT3A* | *DNMT3B* | *DOT1L* | *DPYD* | *DYNC1H1* | *DYNLL1* | *E2F3* |
| *ECT2* | *EED* | *EGF* | *EGFR* | *EMSY* | *EP300* | *EPAS1* | *EPCAM* |
| *EPHA2* | *EPHA3* | *EPHA5* | *EPHA6* | *EPHA7* | *EPHA8* | *EPHB1* | *EPHB4* |
| *ERBB2* | *ERBB3* | *ERBB4* | *ERCC1* | *ERCC2* | *ERCC3* | *ERCC4* | *ERCC5* |
| *ERG* | *ERRFI1* | *ESR1* | *ETV1* | *ETV4* | *ETV5* | *ETV6* | *EWSR1* |
| *EZH2* | *EZR* | *FAM135B* | *FAM46C* | *FANCA* | *FANCC* | *FANCD2* | *FANCE* |
| *FANCF* | *FANCG* | *FANCI* | *FANCL* | *FANCM* | *FARP1* | *FAS* | *FAT1* |
| *FAT3* | *FAT4* | *FBXO31* | *FBXW7* | *FEV* | *FGF1* | *FGF10* | *FGF12* |
| *FGF14* | *FGF18* | *FGF19* | *FGF2* | *FGF21* | *FGF23* | *FGF3* | *FGF4* |
| *FGF5* | *FGF6* | *FGF7* | *FGF9* | *FGFR1* | *FGFR2* | *FGFR3* | *FGFR4* |
| *FGR* | *FH* | *FLCN* | *FLI1* | *FLT1* | *FLT3* | *FLT4* | *FNDC3B* |
| *FOS* | *FOXA1* | *FOXL2* | *FOXO1* | *FRS2* | *FUBP1* | *FUS* | *FYN* |
| *GABRA6* | *GALNT12* | *GATA1* | *GATA2* | *GATA3* | *GATA4* | *GATA6* | *GEN1* |
| *GID4* | *GLI1* | *GLI2* | *GLI3* | *GNA11* | *GNA13* | *GNAQ* | *GNAS* |
| *GREM1* | *GRIN2A* | *GRM3* | *GSK3B* | *H2AX* | *H3-3B* | *H3C2* | *HCK* |
| *HDAC1* | *HDAC2* | *HDAC6* | *HDAC9* | *HGF* | *HMGA1* | *HMGA2* | *HNF1A* |
| *HRAS* | *HSD3B1* | *HSP90AA1* | *HTATIP2* | *ID3* | *IDH1* | *IDH2* | *IDO1* |
| *IGF1R* | *IGF2* | *IKBKB* | *IKBKE* | *IKZF1* | *IL7R* | *INHBA* | *INPP4B* |
| *IRF2* | *IRF4* | *IRS2* | *ITK* | *JAK1* | *JAK2* | *JAK3* | *JAZF1* |
| *JUN* | *KAT6A* | *KDM5A* | *KDM5B* | *KDM5C* | *KDM6A* | *KDR* | *KEAP1* |
| *KEL* | *KLF5* | *KLHL6* | *KMT2A* | *KMT2C* | *KMT2D* | *KNSTRN* | *KRAS* |
| *LCK* | *LIMK1* | *LMO1* | *LRIG1* | *LRP1* | *LRP1B* | *LRP2* | *LTK* |
| *LYN* | *LZTR1* | *MACC1* | *MAF* | *MAGI2* | *MALAT1* | *MAML2* | *MAP2K1* |
| *MAP2K2* | *MAP2K4* | *MAP3K1* | *MAP3K13* | *MAP4K5* | *MAPK1* | *MAX* | *MCF2L* |
| *MCL1* | *MDM2* | *MDM4* | *MECOM* | *MED12* | *MEF2B* | *MEN1* | *MET* |
| *MGMT* | *MITF* | *MKNK1* | *MLH1* | *MLLT3* | *MR1* | *MRE11* | *MS4A1* |
| *MSH2* | *MSH3* | *MSH6* | *MST1R* | *MTAP* | *MTG1* | *MTOR* | *MUC16* |
| *MUTYH* | *MYB* | *MYBL1* | *MYC* | *MYCL* | *MYCN* | *MYD88* | *MYH11* |
| *MYOD1* | *NAB2* | *NBN* | *NCOA2* | *NCOR1* | *NECTIN4* | *NEK11* | *NET1* |
| *NF1* | *NF2* | *NFE2L2* | *NFIB* | *NFKBIA* | *NKX2-1* | *NOTCH1* | *NOTCH2* |
| *NOTCH3* | *NOTCH4* | *NPAT* | *NPM1* | *NR4A3* | *NRAS* | *NRG1* | *NRG3* |
| *NSD1* | *NSD2* | *NSD3* | *NT5C2* | *NTHL1* | *NTRK1* | *NTRK2* | *NTRK3* |
| *NUP88* | *NUP93* | *NUTM1* | *OBSCN* | *P2RY8* | *PAK1* | *PAK3* | *PALB2* |
| *PARP1* | *PARP2* | *PARP3* | *PARP4* | *PAX3* | *PAX5* | *PAX7* | *PBRM1* |
| *PBX1* | *PCA3* | *PDCD1* | *PDCD1LG2* | *PDGFB* | *PDGFRA* | *PDGFRB* | *PDK1* |
| *PHF6* | *PHOX2B* | *PIK3C2B* | *PIK3C2G* | *PIK3C3* | *PIK3CA* | *PIK3CB* | *PIK3CD* |
| *PIK3CG* | *PIK3R1* | *PIK3R2* | *PIM1* | *PKD2* | *PKN1* | *PLA2G1B* | *PLCG2* |
| *PML* | *PMS2* | *POLB* | *POLD1* | *POLE* | *POT1* | *PPARG* | *PPP2R1A* |
| *PRDM1* | *PREX2* | *PRKACA* | *PRKACB* | *PRKAR1A* | *PRKCI* | *PRKDC* | *PRKN* |
| *PRPF38B* | *PRSS1* | *PRSS8* | *PTCH1* | *PTEN* | *PTK2* | *PTK6* | *PTPN11* |
| *PTPRT* | *QKI* | *RAC1* | *RAD21* | *RAD50* | *RAD51* | *RAD51B* | *RAD51C* |
| *RAD51D* | *RAD52* | *RAD54B* | *RAD54L* | *RAF1* | *RANBP2* | *RARA* | *RASA1* |
| *RB1* | *RBBP8* | *RBM10* | *RECQL* | *RECQL4* | *REL* | *RELA* | *RELB* |
| *RET* | *REV3L* | *RGS7* | *RHBDF2* | *RHEB* | *RHOA* | *RICTOR* | *RIT1* |
| *RNASEL* | *RNF43* | *ROCK2* | *ROS1* | *RPTOR* | *RSPO2* | *RSPO3* | *RUNX1* |
| *RUNX1T1* | *RXRA* | *SDC4* | *SDHA* | *SDHAF2* | *SDHB* | *SDHC* | *SDHD* |
| *SERPINB3* | *SERPINB4* | *SETBP1* | *SETD2* | *SF3B1* | *SGK1* | *SHQ1* | *SIK1* |
| *SKP2* | *SLC1A2* | *SLC34A2* | *SLC6A2* | *SLIT2* | *SLX4* | *SMAD2* | *SMAD3* |
| *SMAD4* | *SMARCA2* | *SMARCA4* | *SMARCD1* | *SMARCE1* | *SMO* | *SNCAIP* | *SND1* |
| *SOCS1* | *SOX2* | *SOX9* | *SPEN* | *SPINK1* | *SPOP* | *SPTA1* | *SRC* |
| *SRGAP1* | *SRMS* | *SRSF2* | *SS18* | *SSX1* | *STAG2* | *STAT3* | *STAT4* |
| *STAT6* | *STK11* | *STK24* | *SUFU* | *SUZ12* | *SYK* | *TACSTD2* | *TAF1* |
| *TARBP2* | *TBX3* | *TCF3* | *TCF7L2* | *TEK* | *TENT5C* | *TERC* | *TERT* |
| *TET1* | *TET2* | *TET3* | *TFE3* | *TFEB* | *TGFBR1* | *TGFBR2* | *TIE1* |
| *TIPARP* | *TMEM127* | *TMPRSS2* | *TNFAIP3* | *TNFRSF14* | *TNFRSF19* | *TNFSF11* | *TNFSF13B* |
| *TNK2* | *TOP1* | *TOP2A* | *TP53* | *TP63* | *TPMT* | *TRAF7* | *TRIO* |
| *TSC1* | *TSC2* | *TSHR* | *TSPAN1* | *TSPAN31* | *TYK2* | *TYRO3* | *U2AF1* |
| *UGT1A1* | *USP6* | *VEGFA* | *VGLL3* | *VHL* | *WEE1* | *WEE2* | *WISP3* |
| *WNK1* | *WRN* | *WT1* | *XPO1* | *XRCC2* | *XRCC3* | *YAP1* | *YES1* |
| *YWHAE* | *ZBTB2* | *ZFHX3* | *ZNF217* | *ZNF703* | *ZNF750* | *ZNRF3* | *ZRSR2* |

**Supplemental Table 2. Significantly mutated genes**

| gene | p-value | q-value |
| --- | --- | --- |
| *TP53* | 0 | 0 |
| *SMAD4* | 1.11E-15 | 1.05E-11 |
| *FBXW7* | 3.00E-15 | 1.52E-11 |
| *PIK3CA* | 3.22E-15 | 1.52E-11 |
| *APC* | 4.44E-15 | 1.68E-11 |
| *ACVR2A* | 9.10E-15 | 2.69E-11 |
| *KRAS* | 9.99E-15 | 2.69E-11 |
| *PTEN* | 1.95E-08 | 4.61E-05 |
| *SPTA1* | 5.89E-08 | 1.24E-04 |
| *ATM* | 1.14E-07 | 2.15E-04 |
| *TCF7L2* | 1.98E-07 | 3.39E-04 |
| *EPHB1* | 9.29E-07 | 1.41E-03 |
| *FGF7* | 9.71E-07 | 1.41E-03 |
| *BRAF* | 1.24E-06 | 1.67E-03 |
| *FAM135B* | 1.33E-06 | 1.68E-03 |
| *RNF43* | 3.15E-06 | 3.71E-03 |
| *MAP2K4* | 5.82E-06 | 6.46E-03 |
| *GLI3* | 1.10E-05 | 1.15E-02 |
| *PREX2* | 1.20E-05 | 1.19E-02 |
| *EPHA7* | 1.37E-05 | 1.29E-02 |
| *EPHA3* | 1.54E-05 | 1.38E-02 |
| *ARID1A* | 1.63E-05 | 1.40E-02 |
| *EPHA5* | 2.36E-05 | 1.93E-02 |
| *SMAD2* | 2.84E-05 | 2.23E-02 |
| *CARD11* | 3.59E-05 | 2.71E-02 |
| *NRAS* | 4.97E-05 | 3.61E-02 |
| *FGFR1* | 5.22E-05 | 3.65E-02 |
